# EhVps29 Has a Role in the Location of the Retromer Complex and the Function of Key Virulence Factors in *Entamoeba histolytica*

**DOI:** 10.3390/microorganisms14050976

**Published:** 2026-04-26

**Authors:** Diana Martínez-Valencia, Guillermina García-Rivera, Anel Lagunes-Guillén, Daniel Talamás-Lara, Sarita Montaño, Esther Orozco, Cecilia Bañuelos

**Affiliations:** 1 Departamento de Infectómica y Patogénesis Molecular, Centro de Investigación y de Estudios Avanzados del Instituto Politécnico Nacional (Cinvestav), GAM, Ciudad de México 07360, Mexico; damartinez@cinvestav.mx (D.M.-V.); gugarcia@cinvestav.mx (G.G.-R.); anel.e.lagunes-guillen@uit.no (A.L.-G.); 2 Unidad de Microscopía Electrónica, Laboratorios Nacionales de Servicios Experimentales (LaNSE), Cinvestav, GAM, Ciudad de México 07360, Mexico; dtalamas@cinvestav.mx; 3 Laboratorio de Bioinformática y Simulación Molecular, Universidad Autónoma de Sinaloa, Culiacán 80010, Mexico; mmontano@uas.edu.mx; 4 Programa Transdisciplinario en Desarrollo Científico y Tecnológico para la Sociedad, Departamento de Investigación y Estudios Multidisciplinarios, Cinvestav, GAM, Ciudad de México 07360, Mexico

**Keywords:** *Entamoeba*, ESCRT, Golgi apparatus, recycling, Vps26, Vps29, Vps35

## Abstract

The retromer is a highly conserved complex that mediates the trafficking of cargo proteins to the plasma membrane or the trans-Golgi network. In pathogenic microorganisms, retromer-dependent transport contributes to the delivery of virulence factors and promotes infection. The retromer consists of a sorting nexin dimer (SNX) and a cargo-selection complex (CSC), formed by Vps26, Vps35, and Vps29. In *Entamoeba histolytica*, the parasite that causes human amoebiasis, the retromer functions as a Rab7A GTPase effector and participates in phagocytosis and cytotoxicity. Although we previously characterized the roles of EhVps26 and EhVps35, the function of EhVps29 remained unclear. In this study, we analyzed the subcellular localization and functional role of EhVps29 in adhesion, phagocytosis, and cytopathic effect. EhVps29 localized to the plasma membrane, cytosol, vesicles, tubules, Golgi-like structures, MVBs and, for the first time, the nucleus. Immunofluorescence and Western blot assays demonstrated that EhVps29 modulates the localization of EhVps26, EhADH adhesin, and EhCP112 cysteine protease. *Ehvps29* gene silencing and overexpression confirmed its involvement in virulence-associated processes. Immunoprecipitation and confocal microscopy results showed the interaction among EhVps29 and the ESCRT machinery members EhVps36 and EhADH. Our results indicate that EhVps29 is involved in parasite virulence and protein trafficking through recycling or degradation pathways.

## 1. Introduction

Endocytosis is a highly dynamic process in which endosomes function as sorting organelles. In the endosomal membrane, multiple domains coexist, leading to membrane remodeling that results in tubulation or fission events during protein sorting towards the recycling pathways or the *trans*-Golgi network (TGN) in retrograde transport [1]. The equilibrium between cargo protein degradation and recycling is essential for cellular homeostasis. In the recycling pathway, a well-known and widely conserved complex is the retromer [2,3], which transports a broad variety of molecules, including the cation-independent mannose-6-phosphate receptor [3], the SorLA receptor, a precursor related to Alzheimer’s disease [4], the Wntless protein in *Caenorhabditis elegans* and *Drosophila melanogaster* [2,5], and the GLR1 glutamate receptor in *C. elegans* [6]. In addition, some virulence factors have also been identified as retromer targets in pathogenic protozoans, e.g., the SORTL/SORTLR (sortilin receptor) from *Plasmodium falciparum* [7] and *Toxoplasma gondii* [8,9], and the ISG65 and ISG75 transmembrane proteins from *Trypanosoma brucei* [10].

The retromer complex is formed by two sub-complexes: the sorting nexin dimer (SNX) and the cargo-selecting complex (CSC). The SNX subcomplex is formed by the Vps5/Vps17 dimer in yeast [11] or by PX-BAR proteins, such as SNX1/2 and SNX5/6, in higher eukaryotes [12,13]. The main function of SNX proteins is to detect membrane lipids and create arrays of tubular subdomains for cargo sorting [14,15]. Meanwhile, the CSC is constituted by Vps26, Vps35, and Vps29 proteins [3,16]. Vps26 and Vps35 are involved in cargo identification [17]. Vps26 brings specificity for cargo recognition, depending on the interacting site [18], and it is also important to maintain the Golgi apparatus morphology in eukaryotic cells [19]. Moreover, Vps35 functions as a scaffold [18], and Vps29 stabilizes the CSC [20]. Vps29 promotes CSC detachment from the endosomal membrane through the interaction with the TBC1D5 Rab7 regulator [21,22] and VARP, a Rab21 GEF and Rab32 effector [23,24]. Vps29 is also important for the expression of Vps35 and Vps26, but not for the CI-M6PR retromer cargo [25]. Moreover, it was recently described that Vps29 is also a member of the retriever, a retromer-like complex involved in protein transport from endosomes to the plasma membrane [26].

In *Entamoeba histolytica*, the causative agent of human amoebiasis, the CSC is an effector of the Rab7A GTPase [27], and the Vps29 protein (EhVps29) and Vps26 protein (EhVps26) are both involved in the cytotoxic damage caused by cysteine proteases [27,28]. In addition, our group has reported that EhVps26 and EhVps35 proteins are involved in phagocytosis and protein transport [29,30].

Since the retromer proteins, especially Vps29, regulate the function of other proteins in several biological models, we hypothesized a putative role for EhVps29 as a molecular bridge among certain *E. histolytica* proteins involved in virulence. One possible interaction could be with components of the endosomal sorting complexes required for transport (ESCRT), involved in cargo selection and transport for lysosomal degradation.

In *Arabidopsis thaliana*, the interaction between ESCRT and retromer proteins has been described: an ESCRT-accessory protein (ALIX) regulates the recycling of vacuolar sorting receptors through retromer interaction [31]. Moreover, the EGFR recycling to the plasma membrane is coordinated by the interaction among Tsg101 (ESCRT-I), SNX2 (retromer), and BLOS1 (a protein related to lysosomal degradation) [32]. Furthermore, Vps27 (ESCRT-I) and SNX3 (retromer) interact in the endosomes, leading to domain formation for protein degradation or recycling [33]. In *E. histolytica,* EhADH (an ALIX family protein) is a virulence factor recycled by the retromer through the interaction with EhVps35 [30].

Here, we cloned and expressed the *Ehvps29* gene and produced specific α-EhVps29 antibodies to obtain supplementary insights regarding EhVps29’s location and function in trophozoites. By confocal and transmission electron microscopy approaches, EhVps29 was found in Golgi-like structures and, interestingly, in extracellular vesicles, multivesicular bodies (MVB) and the nucleus of trophozoites. Confocal microscopy and immunoprecipitation assays revealed interactions of EhVps29 with the EhVps36 (ESCRT-II) and EhADH (ESCRT-accessory) proteins. *Ehvps29*-knocked-down trophozoites showed lower rates of adhesion, phagocytosis, and cytopathic effect and exhibited a lesser expression and an atypical localization of the EhCP112, EhADH and EhVps26 proteins. The EhVps26 and EhVps29 co-localization was also diminished in Golgi-like structures.

Overall, our results highlight novel contributions of EhVps29 in parasite virulence and, importantly, its putative role as a molecular bridge that connects degradation and recycling pathways, possibly allowing proteins to reach their fate in an orchestrated manner.

## 2. Materials and Methods

### 2.1. Plasmid Construction and Recombinant Protein Production

cDNA was obtained from an *E. histolytica* RNA library with the first-strand cDNA synthesis kit (Sigma-Aldrich, Vilnius, Lithuania), following the manufacturer’s instructions. To produce the His-tagged recombinant EhVps29 protein and plasmids for overexpression, the complete open-ready frame of the *Ehvps29* gene was PCR-amplified using the following primers: GGTACCATGCTTGTACTTGTTATTGGAG (sense) and GGATCCTATTGTTGTTGTTTCTTATTGAA (antisense). The underlined nucleotides correspond to *KpnI* and *BamHI* restriction sites, respectively. For gene knockdown, the first 400 bases of the *Ehvps29* gene were amplified by PCR using the primers GGTACCATGCTTGTACTTGTTATTGGAGA (sense) and CTCGAGGCACTACCTGGATTAAGGA (antisense), where underlined sequences are *SacI* and *KpnI* recognition sites. The *Ehvps29* gene was subcloned in the pJet1.2/blunt vector (ThermoFisher, Waltham, MA, USA) and cloned in the appropriate sites of pCold-I (Takara Bio, Inc., Shiga, Japan) or pNeo plasmids [34] and the first *Ehvps29* bp within the L4440 vector (Addgene, Watertown, MA, USA). The obtained constructs were named pCold-*Ehvps29*, pNeo-*Ehvps29* and L4440-*Ehvps29*, respectively. The production of the recombinant EhVps29 protein (rEhVps29) was induced in transformed *Escherichia coli* BL21 (DE3) by the addition of 100 µM isopropyl-β-thiogalactoside (IPTG).

### 2.2. α-EhVps29 Polyclonal Antibodies Generation

rEhVps29 was purified with the Hispur Cobalt Resin (Thermo Fisher, Waltham, MA, USA) following the manufacturer’s instructions. An initial dose of 150 µg of purified rEhVps29 was emulsified in Titer-Max Gold (Sigma-Aldrich, Vilnius, Lithuania) and subcutaneously and intramuscularly inoculated in male Wistar rats (a dose per animal). Two more 75 µg doses of rEhVps29 per animal were administered at intervals of 14 days. After 42 days from the initial dose, the animals were bled to obtain antibodies directed to EhVps29. Serum was obtained before each immunization. The rats were kept at the Cinvestav vivarium and constantly monitored for disease signs during the immunization period. The rats were humanely anesthetized before the final bleeding.

### 2.3. E. histolytica Cultures

Trophozoites of *E. histolytica* (strain HM1:IMSS) were axenically cultured in TYI-S-33 medium [35] supplemented with adult bovine serum (BSA) (Equitech-Bio, Inc., Kerrville, TX, USA) at 37 °C and harvested at logarithmic growth phase by an ice bath.

### 2.4. Western Blot Assays

*E. histolytica* trophozoites were lysed by the addition of protease inhibitors (PMSF 100 mM, benzamidine 100 mM, aprotinin 10 mg/mL, pepstatin 1 mg/mL, leupeptin 10 mg/mL and E-64 1 mg/mL) and a freeze–thaw cycle. Cultures of induced *E. coli* BL21 (DE3) bacteria transformed with the pCold-*Ehvps29* construct were lysed with protease inhibitors too; for detecting the histidine tag, we used purified rEhVps29, as described. Additionally, 35 µg of amoebic and bacterial total extracts or 10 µg of purified rEhVps29 were separated by 15% SDS-PAGE, transferred to nitrocellulose filters, and probed with rat α-EhVps29 polyclonal antibodies (1:500), mouse α-EhADH (1:2000) [36], rabbit α-EhCP112 (1:2000) [37], rabbit α-EhVps36 (1:500) [38], rabbit α-EhVps26 (1:500) [29], monoclonal mouse α-actin (1:1000, kindly donated by Dr. Manuel Hernández from Cinvestav), or mouse α-histidine (1:500, Abcam, Waltham, MA, USA) overnight (ON) at 4 °C. Membranes were then washed and incubated with the corresponding α-rat, α-rabbit, or α-mouse-HRP-labeled antibodies (Zymed, 1:10,000, Waltham, MA, USA). Signals were developed with the ECL Prime Western Blotting Detection reagent (G&E Healthcare, Anaheim, CA, USA). Actin was used as an internal loading control.

### 2.5. Confocal Microscopy

For indirect immunofluorescence (IF) assays, we used trophozoites grown in glass coverslips, fixed with 4% paraformaldehyde, permeabilized with 0.5% Triton X-100 (Sigma, Vilnius, Lithuania), blocked with 1% BSA in PBS, and incubated for 1 h with rat α-EhVps29 (1:50), rabbit α-EhVps26 (1:100), rabbit α-EhADH (1:100), rabbit α-EhCP112 (1:50), or rabbit α-EhVps36 polyclonal antibodies at 37 °C. After this time, samples were washed and incubated with α-rabbit or α-rat FITC-coupled secondary antibodies (1:100, Zymed, Waltham, MA, USA), CFL 647 (1:200, Santa Cruz, Dallas, TX, USA) or Alexa 405 (1:200, Abcam, Waltham, MA, USA). Finally, samples were washed and mounted with VECTASHIELD mounting medium (Vector Labs, Newark, CA, USA). We used the NBD C6 ceramide probe (ThermoFisher, Waltham, MA, USA) as a Golgi apparatus marker, according to the manufacturer’s guide. Samples were analyzed with a Carl Zeiss LSM 700 confocal microscope and processed with the Zen Black software (Zeiss; Jena, Germany, 2012). For co-localization analysis, we used the JACoP plugin from the ImageJ software version 1.53t (24 August 2022) [39]. In all cases, we considered 0.5 µm z-stacks of complete cells or selected areas, taking 20 cells per sample.

### 2.6. Transmission Electron Microscopy (TEM)

Trophozoites at steady state or 30 min of erythrophagocytosis were fixed with 2.5% (*v*/*v*) glutaraldehyde in 0.1 M sodium cacodylate buffer, pH 7.2, for 60 min. Then, they were post-fixed for 60 min with 1% (*w*/*v*) osmium tetroxide in the same buffer. After dehydration with increasing concentrations of ethanol and propylene oxide, samples were embedded in Polybed epoxy resins and polymerized at 60 °C for 24 h. Thin sections (60 nm) were contrasted with uranyl acetate and lead. For gold immunolabeling experiments, trophozoites were fixed with 4% PFA and 0.5% glutaraldehyde in PBS for 1 h at RT. Samples were embedded in LR White resin (London Resin Co., Enfield, UK) and polymerized under UV at 4 °C ON. Thin sections were incubated ON with rat α-rEhVps29 (1:20), rabbit α-EhVps26 (1:20) and rabbit α-GM130 (GeneTex, Irvine, CA, USA) (1:20) antibodies, then, with α-rat antibodies conjugated to 10 nm and 15 nm gold particles, and with α-rabbit antibodies conjugated to 30 nm gold particles (Ted Pella Inc., Redding, CA, USA) (1:60). Samples were observed with the JEM-1400 and JEM-1011 (JEOL, Tokyo, Japan) transmission electron microscopes.

### 2.7. Ehvps29 Knockdown Trophozoites

To knock down the *Ehvps29* gene in trophozoites, we used the methodology previously described [40]. Briefly, we transformed competent RNAse III-deficient *E. coli* HT 115 bacteria (rnc14::ΔTn10) with the pL4440-*Ehvps29* construct, and cultures were grown in LB agar supplemented with ampicillin (100 mg/mL) and tetracycline (10 mg/mL). Positive colonies were verified by PCR. After induction of dsRNA, DNAse I (Invitrogen, Waltham, MA, USA) and RNAse A (Ambion, Waltham, MA, USA) were added to remove the remaining dsDNA and ssRNA. The integrity of dsRNA was analyzed in 2% agarose gels. Then, 5 μg/mL of purified dsRNA was added to the amoebae culture (1 × 10^5^) in TYI-S-33 medium. The higher knockdown of the *Ehvps29* gene was observed at 48 h and verified by Western blot and confocal microscopy. Wild-type amoebae grown without added dsRNA were used as a control.

### 2.8. Overexpression of the Ehvps29 Gene in Trophozoites

*E. histolytica* trophozoites (3 × 10^5^) were transfected with 20 µg of the Neo-*Ehvps29* construct or the empty Neo vector as described by Ávalos-Padilla et al. [41], using the SuperFect Transfection Reagent (QIAGEN, Germantown, MD, USA), and following the manufacturer’s instructions. Cultures were grown in M199 media supplemented with 25% BSA. Overexpression was induced by the addition of 5 µg/mL of G-418 (GIBCO, Waltham, MA, USA), a neomycin analog, to the media culture. The overexpression was confirmed by Western blot and IF assays.

### 2.9. Adhesion Assays

Additionally, 1.25 × 10^5^ trophozoites were placed in conic tubes and maintained on ice to interact with human red blood cells (RBCs) at a 1:25 ratio (amoeba:RBCs). After 2, 5, 15 or 30 min of interaction, the reaction was stopped by the addition of 2.5% glutaraldehyde. Adhered erythrocytes were counterstained with 2 mg/mL 3-3’ diaminobenzidine (Sigma, Vilnius, Lithuania) according to Novikoff et al. [42]. Three independent experiments in duplicate were done.

### 2.10. Phagocytosis Assays

Trophozoites (5 × 10^5^) were incubated with human RBCs at a 1:25 ratio for 2, 5, 15 and 30 min at 37 °C. In the case of knocked-down cells, we allowed the interaction for 5 min (pulse). Non-ingested RBCs were lysed by the addition of sterile water and the interaction was continued for 15 and 25 additional min (chase). Then, trophozoites were incubated in TYI medium at 37 °C. For overexpressing trophozoites, the interaction was allowed for 5, 15, 30, 60 and 90 min. At each point, trophozoites were fixed and processed for IF or lysed for Western blot assays, as previously described. Samples were processed to counterstain the ingested erythrocytes with diaminobenzidine. Three independent experiments in duplicate were done.

### 2.11. Cytopathic Effect of Trophozoites on Cell Monolayers

Confluent MDCK cell monolayers were incubated with 10^5^ trophozoites per well at 37 °C until wild-type control trophozoites destroyed ~80% of the monolayers (40 min). Then, epithelial cells were washed to remove adhered trophozoites, fixed with 2.5% glutaraldehyde, and stained with 1% methylene blue. The color was extracted from the remaining monolayers to measure the optical density at a fixed wavelength of 660 nm. MDCK cells incubated in TYI medium were used as integrity control. Two independent experiments in quadruplicate were done.

### 2.12. Immunoprecipitation

Immunoprecipitation assays were made by the interaction of 200 μL of protein G-agarose (Invitrogen, Waltham, MA, USA) and α-EhVps29 antibodies or preimmune serum ON at 4 °C. Trophozoites at steady state or 15 min of erythrophagocytosis were lysed in the presence of the same protease inhibitors used for Western blot, 10 mM Tris-HCl and 50 mM NaCl, using freeze-thawing cycles. Amoebic TE were cleared by incubation with 50 μL of protein G-agarose and then used to immunoprecipitate proteins by interaction with α-EhVps29 or preimmune serum coupled to the resin for 2 h at 4 °C. Immunoprecipitated proteins were revealed and analyzed by Western blot assays with α-EhADH, α-EhVps26, α-EhVps36 and α-EhVps29 antibodies.

### 2.13. Data Analysis and Statistical Methods

For all assays, values were expressed as the mean ± standard error of three independent experiments unless another condition was stated. Plots and statistical analysis were done using Graphpad Prism version 6.0 for Windows (GraphPad Software, La Jolla, CA, USA). Statistical analyses were made using unpaired t-student tests, but if comparing more than two groups, we used one-way ANOVA with Dunnett’s post-test. *p* values are described in each figure.

### 2.14. Three-Dimensional Models

EhADH and EhCP112 tridimensional models were obtained from previous work [43]. The EhVps29 structure corresponds to the crystal experimentally obtained by Srivastava et al. [28] (RCSB: 5XCE:A), and the EhVps36 structure corresponds to the previously reported one by Díaz-Hernández et al. [38].

### 2.15. Molecular Docking

Average stabilized structures of EhADH, EhCP112, EhVps29 and EhVps36 were used for molecular docking at the ClusPro 2.0 server [44,45], and the best predicted interaction was selected according to the lowest energy and members’ number. Docking structures were refined in the Firedock server [46,47] and analyzed using the PDBsum server [48]. Graphs were obtained with the VMD software version 1.9.4 LATEST ALPHA (2023-06-08), last accessed on 21 February 2025 (http://www.ks.uiuc.edu/Research/vmd/) [49].

## 3. Results

### 3.1. EhVps29 Is Present at Trophozoite Plasma Membrane, Cytosol, Vesicles, Tubular Structures, Extracellular Vesicles, and Nucleus

Despite the little evidence about protozoan Vps29 protein function, it is known that *E. histolytica* Vps29 (EhVps29, access number EHI_025270) has a metallophosphatase fold and a role in phagocytosis and cytotoxic damage [28].

To deepen the characterization of the EhVps29 protein function, our first step was to clone the full *Ehvps29* gene in the pCold I plasmid to obtain a his-tagged EhVps29 recombinant protein (rEhVps29) and then generate specific α-EhVps29 polyclonal antibodies in rats.

**Figure 1:**
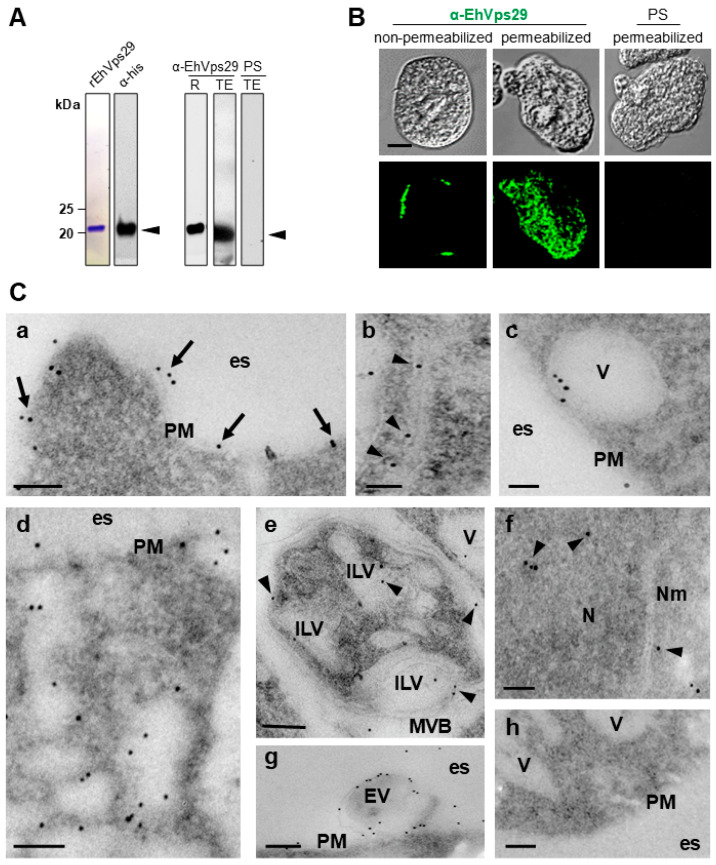
**Expression and localization of EhVps29.** (**A**) Immunodetection of EhVps29. The purified recombinant EhVps29 protein (rEhVps29) detected by Coomassie staining and the α-his antibody. The recombinant protein (R) and *E. histolytica* total extracts (TE) were probed with the α-EhVps29 polyclonal antibody. PS: preimmune serum. Numbers at left indicate molecular weights. (**B**) Indirect immunofluorescence of non-permeabilized and permeabilized trophozoites. EhVps29 was detected using the α-EhVps29 antibody, followed by the FITC-coupled secondary antibodies. (**C**) Ultrastructural localization of EhVps29 by TEM. Ultrathin sections were incubated with α-EhVps29 or preimmune serum, and 10 nm gold-labeled α-rat antibodies. Localization of EhVps29 in plasma membrane (**a**), double-membrane tubular structures (**b**), vesicles (**c**–**e**), MVBs and ILVs (**e**), nucleus (**f**), extracellular vesicles (**g**). Negative control, trophozoite sectionsonly incubated with secondary antibodies (**h**). Scale bars: 100 nm. PM: Plasma membrane. V: Vesicles. es: Extracellular space. MVB: Multivesicular body. ILV: Intraluminal vesicle. EV: Extracellular vesicle. N: nucleus. Nm: nucleus membrane.

Through Western blot assays, both the α-his and α-rEhVps29 antibodies detected a 21-kDa band in transformed bacteria lysates (Figure 1A). This weight corresponds to EhVps29 (20 kDa) plus the 6x-his tag. In amoebic lysates, a 20-kDa band was visible, while no bands were observed by using the preimmune sera (Figure 1A).

To determine the EhVps29 cellular location, we used fixed permeabilized and non-permeabilized amoebae and the α-EhVps29 antibodies for immunofluorescence experiments. By confocal microscopy, this protein was detected in the outer face of the plasma membrane in non-permeabilized trophozoites and in the cytosol of permeabilized cells (Figure 1B). In previous works, EhVps26 and EhVps35 have also been detected in the outer plasma membrane [29,30].

TEM images of ultrathin sections of trophozoites treated with α-EhVps29 antibodies and then with α-rat gold-coupled secondary antibodies confirmed these results. Gold particles appeared in plasma membrane (Figure 1C, panel a), in 300 nm double-membrane tubular structures (Figure 1C, panel b), in cytosol and vesicles (Figure 1C, panels c–e), in intraluminal vesicles (ILV) (Figure 1C, panel e), multivesicular bodies (MVBs) (Figure 1C, panel e), and, intriguingly, in the nucleus (Figure 1C, panel f). Finally, we also detected EhVps29 in extracellular vesicles (Figure 1C, panel g). The tubular structures observed by TEM resemble the tubules formed by the retromer in other species [50]. The localization of EhVps29 in extracellular vesicles has also been reported [51]. Although no reports have documented the presence of the Vps29 protein in the nucleus, the retromer-associated protein SNX11 has been involved in the nuclear translocation of the factor II receptor-like 1 (F2rl1) [52]. Moreover, a calcium-binding protein in *E. histolytica*, EhCaBP6, has been localized to the nucleus, implicated in cell proliferation; notably, its nuclear translocation has been shown to be calcium-dependent, while no nuclear localization signal is present [53].

Furthermore, the possibility that the CSC is preassembled in the cytosol before its translocation into the nucleus cannot be excluded. In such a scenario, the nuclear pore complex permits the import of molecules up to approximately 39 nm in length [53]. In *Amoeba proteus*, however, nuclear pores have been shown to accommodate the passage of larger molecules, with sizes ranging from 60 to 80 nm [54]. Given that the human CSC has an approximate length of 21 nm [55], these observations suggest that nuclear translocation of the *E. histolytica* retromer complex is structurally feasible.

Alternatively, we investigated the possibility that CSC proteins present in the nucleus could be performing some functions. In this context, nuclear export signals (NESs) were analyzed in EhVps26, EhVps35, and EhVps29 using the LocNES server [56]. The analyses identified at least five NES motifs in EhVps26 (EHI_062490), ten in EhVps35 (EHI_002990), and one in EhVps29 (EHI_025270), all with scores ≥ 0.2. These findings do not exclude the possibility that CSC proteins, including EhVps29, transiently localize to the nucleus to fulfill specific functions and are subsequently exported to the cytosol under conditions that remain to be defined.

The results described above confirm that EhVps29 is located in organelles where the retromeric EhVps26 and EhVps35 proteins have been observed [27,28,29,30,57], reinforcing the conserved role of retromer proteins in *E. histolytica* and opening novel questions regarding functions not yet studied.

### 3.2. EhVps29 Is in Golgi-like Structures

In higher eukaryotes, it is well known that the *trans* face of the Golgi apparatus (TGN) is a target organelle for protein recycling by the retromer [3,58,59]. Previous works demonstrated the location of EhVps26 in Golgi-like structures [29,60]. Here, to investigate if EhVps29 also resides in the Golgi apparatus, we used α-EhVps26 [29] and α-EhVps29specific antibodies and the NBD-C6 ceramide probe, previously used as a Golgi marker in *E. histolytica* [61,62].

**Figure 2:**
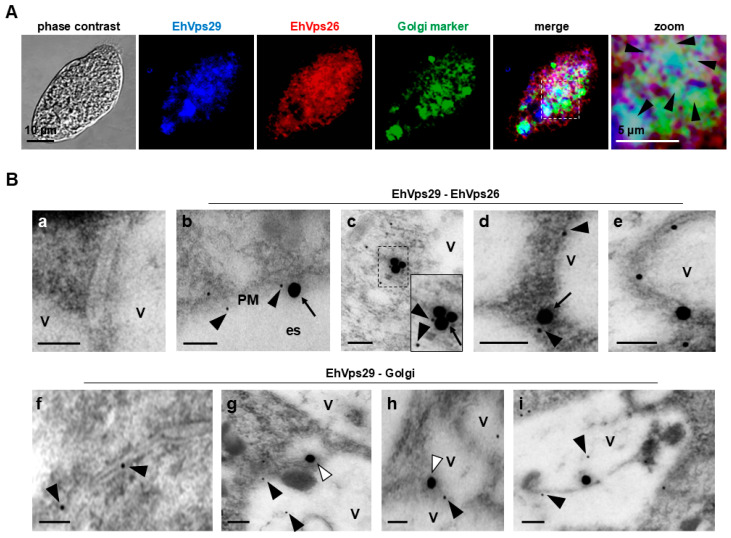
**Localization of EhVps26 and EhVps29 in the Golgi-like structures.** (**A**) EhVps29 (blue), EhVps26 (red), and the Golgi-like structures (green) were detected through confocal microscopy. Fixed and permeabilized *E. histolytica* trophozoites were incubated with the antibodies and then with a fluorescence-labeled secondary antibody. Arrowheads: Areas where EhVps26 and EhVps29 are co-localizing in the Golgi. (**B**) Detection of EhVps29, EhVps26, and the Golgi by TEM. First, we used rat α-EhVps29 and rabbit α-EhVps26 antibodies, followed by gold-coupled secondary antibodies. Then, we used rat α-EhVps29 and rabbit α-GM130 as primary antibodies, followed by α-rat (15 nm) and α-rabbit (30 nm) gold-coupled secondary antibodies in ultrathin trophozoite sections. (**a**) Negative control. (**b**) Localization of EhVps26 and EhVps29 in the plasma membrane. (**c**) EhVps26 and EhVps29 retromeric proteins in the cytosol. (**d**,**e**) EhVps29 and EhVps26 in vesicle membranes. (**f**) Localization of EhVps29 in cisternae-like structures. (**g**–**i**) EhVps29 and Golgi marker in vesicle membranes and within vesicles. PM: Plasma membrane. es: Extracellular space. V: Vesicles. Black arrowheads: EhVps29. Arrows: EhVps26. White arrowheads: cis-Golgi marker. Scale bars: 100 nm.

Via confocal microscopy assays, EhVps29 and EhVps26 proteins were found in the cytosol and vesicles, co-localizing with the Golgi marker (Figure 2A), strongly suggesting the putative assembly of the retromer in this organelle.

We performed TEM immunoassays, using the α-EhVps29 and α-EhVps26 polyclonal antibodies, followed by α-rat and α-rabbit gold-coupled secondary antibodies, respectively. In these experiments, we also used the α-GM130 antibody, a specific marker for the Golgi-apparatus *cis* face [62]. TEM micrographs showed the EhVps29 and EhVps26 proteins close to each other in the plasma membrane (Figure 2B, panel b), cytosol (Figure 2B, panels c and d), and vesicle membranes (Figure 2B, panels d and e). The proximity of EhVps29 and EhVps26 suggests the assembly of the CSC in these organelles.

In addition, we found EhVps29 close to the Golgi marker in structures resembling Golgi cisternae (Figure 2B, panel f), in vesicle membranes (Figure 2B, panels g and h) and inside vesicles (Figure 2B, panel i). Given that Vps29 proteins could be present in both the retromer and retriever, we cannot rule out that some EhVps29 particles could correspond to one complex or another. Further studies are needed to prove the existence of the retriever complex in *E. histolytica*. However, our results support the localization of CSC components in Golgi-like structures.

### 3.3. During Phagocytosis, EhVps29 Is Mobilized to Phagocytic Cups, Phagosomal Membranes, and Double-Membrane Tubular Structures

**Figure 3:**
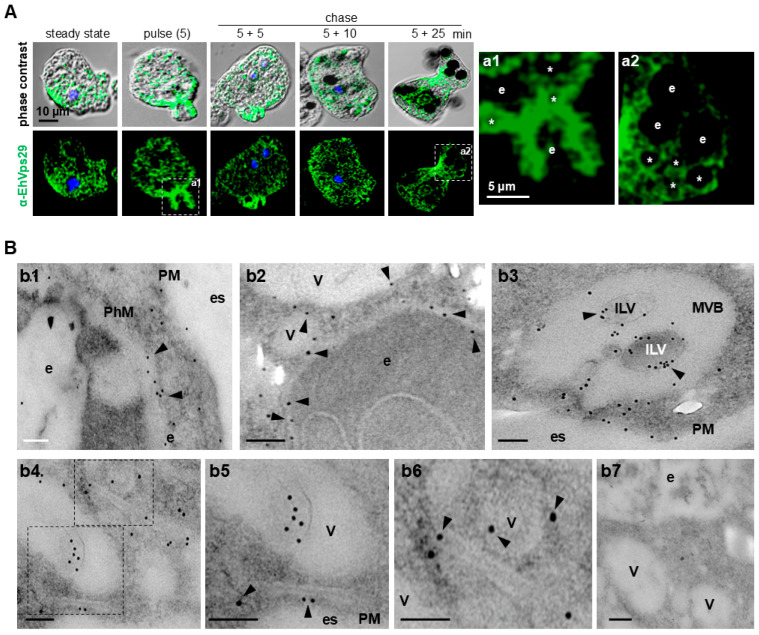
**Localization of EhVps29 during phagocytosis.** (**A**) Confocal immunofluorescence images of trophozoites at different times of phagocytosis. Steady state (0 min), pulse (5 min) and pulse and chase (5 + 5, 5 + 10 and 5 + 25 min). At right: Zoom areas marked in (**a1**,**a2**). e: Erythrocyte. *: Vesicles adjacent to phagosomes decorated by EhVps29. (**B**) Ultrastructural localization of EhVps29 by TEM. Trophozoites at 30 min of erythrophagocytosis treated with the α-EhVps29 antibody, followed by 10 nm gold-labeled α-rat antibody. (**b1**,**b2**) EhVps29 in phagosomal membranes. (**b3**) MVBs containing EhVps29. (**b4**) EhVps29 in enlarged double-membrane structures and zoom to marked areas in squares (**b5**,**b6**). (**b7**) Negative control. Scale bars: 200 nm. e: Erythrocyte. PM: Plasma membrane. es: Extracellular space. PhM: Phagosomal membrane. V: Vesicle. MVB: Multivesicular body. ILV: Intraluminal vesicle.

Endocytosis is the cellular process in which the retromer sorts and facilitates cargo transport from the endosomal membrane to a target organelle to maintain cellular homeostasis [63]. In this work, we used erythrophagocytosis as a model to study the role of EhVps29 in this pathogenic event. Wild-type trophozoites from the HM1:IMSS strain were stimulated with human red blood cells (RBCs) in pulse and chase experiments, as described in the Section 2.10, to discriminate non-ingested RBCs. Confocal microscopy results confirmed that at steady state, EhVps29 is distributed through the amoebic cytosol (Figure 3A). After RBC stimulation, it is mobilized to the phagocytic cups during the pulse (5 min) and early chase times (5 + 5 min) (Figure 3A). Then, at 5 + 10 and 5 + 25 min of pulse and chase, the protein was observed around phagosomes and in vesicles near them (Figure 3A, asterisks). We hypothesize that the amoebic retromer could be there for sorting cargoes and forming recycling domains [2,26,64], either to transport proteins to the Golgi apparatus or directly to the plasma membrane. Early transport of cargoes from endosomes to the plasma membrane could be mediated in *E. histolytica* by SNX3-like proteins, as in higher eukaryotes [2,64], or by a hypothetical retriever complex, since Vps29 is part of both complexes [20,26].

To extend the study of EhVps29 during phagocytosis, we performed TEM assays using α-EhVps29 and gold-coupled α-rat antibodies. Our results confirmed the location of EhVps29 in the phagosome membranes and RBC-adjacent vesicles (Figure 3B, panels b1 and b2). EhVps29 was also found in ILVs of MVBs (Figure 3B, panel b3), which suggests that EhVps29 transport could be mediated by the ESCRT machinery towards degradation, a role for EhVps29 in protein sorting in the MVB pathway or other functions not yet described. We also noticed the presence of EhVps29 in enlarged structures near vesicles (Figure 3B, panels b4–b6).

Taken together, these results point out the participation of EhVps29 in phagocytosis, probably for selecting cargo proteins for their transport to target organelles.

### 3.4. EhVps29 and ESCRT Components Interact During Phagocytosis

Endosomes are key for cargo sorting and transport [65] towards recycling or degradation [66], in which the retromer and ESCRT complexes are the two major representatives. The equilibrium between these pathways is crucial for cellular homeostasis, as the disruption of any of them has been associated with pathogenic conditions [65,67,68]. In *A. thaliana*, the co-localization of ALIX (an ESCRT-accessory protein) and retromeric Vps26 and Vps29 proteins [31] suggested an interaction between ESCRT and retromer components.

It is worth noting that in the TEM micrographs of this work, we observed the presence of EhVps29 in MVBs (Figure 1C, panel e; Figure 3B, panel b3). This and previous findings [30] led us to hypothesize a functional relationship between proteins of the retromer and ESCRT complexes in *E. histolytica*.

**Figure 4:**
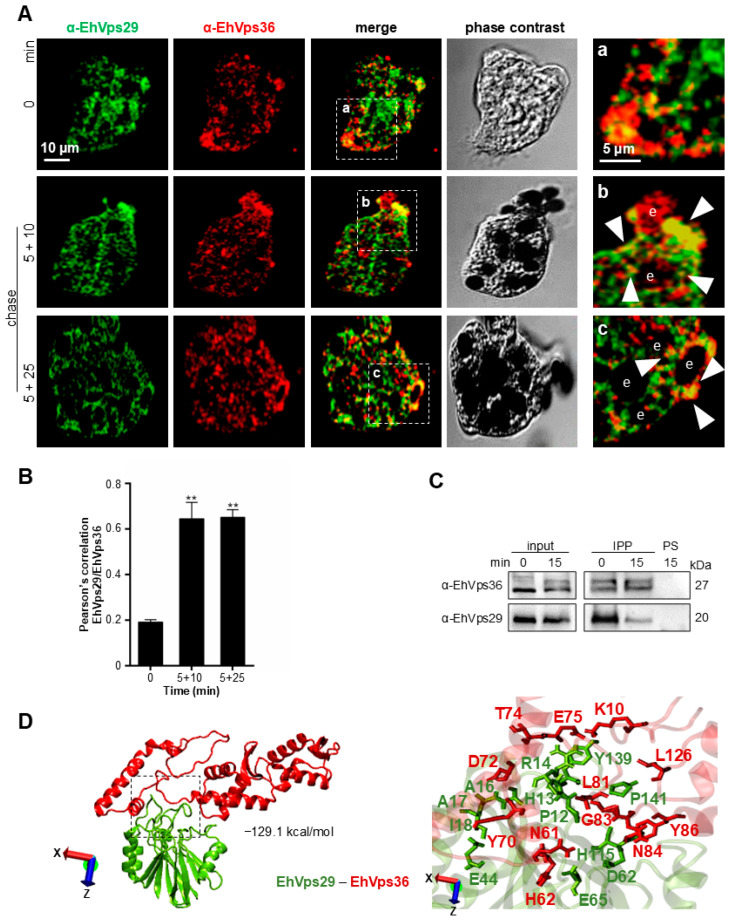
**Interaction of EhVps29 with EhVps36 (ESCRT-II) during phagocytosis.** (**A**) Confocal microscopy detecting EhVps29 (green) and EhVps36 (red) in trophozoites at 0 and 5, 5 + 10 and 5 + 25 min of phagocytosis. Images at the bottom (**a**–**c**) indicate selected areas in merged images. Arrowheads: EhVps29 and EhVps36 co-localization. e: Erythrocyte. (**B**) Pearson’s correlation between EhVps29 and EhVps36 co-localization. A total of 20 z-stacks of 0.5 µm were considered for quantification. ** *p* < 0.01. (**C**) Trophozoite total extracts were immunoprecipitated using α-EhVps29 antibodies at 0 or 15 min or phagocytosis. The immunoprecipitated proteins were separated by 15% SDS-PAGE and bands were detected with α-EhVps29 and α-EhVps36 antibodies. PS: Preimmune serum. Numbers at right show molecular weights. (**D**) Molecular docking of EhVps29 and EhVps36 performed by blind docking assays in the ClusPro server. Right: Zoom of the region marked in the square, containing the contact surface between proteins (for further details see Table 1). Images were obtained by the VMD software version 1.9.4 LATEST ALPHA (2023-06-08). Binding energy is indicated.

In this work, we chose EhVps36 (an ESCRT-II member) [38] and the EhADH adhesin (an ALIX family member and ESCRT-accessory protein) [69] as representative components of the early and late ESCRT pathways, respectively. We aimed to investigate the interaction between EhVps29 and these ESCRT components during erythrophagocytosis. Our first approach was to use the α-EhVps29 and α-EhVps36 antibodies and their corresponding secondary antibodies to detect the proteins by confocal microscopy in pulse and chase phagocytosis assays. The results showed EhVps29 and EhVps36 localizing in the cytosol at steady state, whereas both proteins were redirected to the phagocytic cups and phagosomes after RBC stimulus (Figure 4A). Low co-localization areas appeared at steady state, showing a low Pearson’s correlation (0.2). The values increased to 0.65 at 5 + 10 min and to 0.67 at 5 + 25 min of pulse and chase, evidencing a higher co-localization of the retromer and ESCRT complexes at late phagocytosis times (Figure 4B).

The interaction between retromer and ESCRT components was corroborated by co-immunoprecipitation assays, using α-EhVps29 antibodies coupled to G-agarose beads, interacting with amoebic total extracts. Western blot assays using α-EhVps36 antibodies revealed a 27-kDa band, corresponding to the molecular weight of EhVps36. EhVps36 immunoprecipitation was observed at steady state and at 5 + 10 min of phagocytosis. As expected, EhVps29 appeared as a 20-kDa band (Figure 4C).

To get further insights regarding the putative interaction sites and bond conformations between EhVps29 and EhVps36, we performed molecular docking assays in the ClusPro server, using the EhVps29 crystal (RCSB: 5XCE) [28] and the EhVps36 predicted and refined structures [38]. The predicted EhVps29 and EhVps36 interaction had a strength of −129.1 kcal/mol (Figure 4D), mediated by the H13, R14, H88, W93, H115, T116, K118, L119, and Y139 of EhVps29 and the R160, D161, E165, N169, E208, T228, Q229, L231, Q232, Y233, and Y235 residues of EhVps36 (Figure 4D, Table 1). We found thirteen hydrogen bonds and one salt bridge in this interaction (Table 1).

Overall, our results strongly suggest that the interaction of retromer (EhVps29) and ESCRT-II (EhVps36) complexes increases during phagocytosis, as evidenced by confocal microscopy and Pearson’s correlation results. Further studies will be necessary to determine if this interaction is direct or mediated by other proteins and to elucidate its contribution to *E. histolytica* virulence.

### 3.5. EhVps29 Interacts with the EhADH Adhesin

To continue with the characterization of components of the retromer–ESCRT systems, we performed pulse and chase erythrophagocytosis experiments and processed the samples for immunofluorescence assays, now using the α-EhVps29 and α-EhADH [36] primary antibodies and α-rat and α-rabbit fluorochrome-coupled secondary antibodies, respectively.

**Figure 5:**
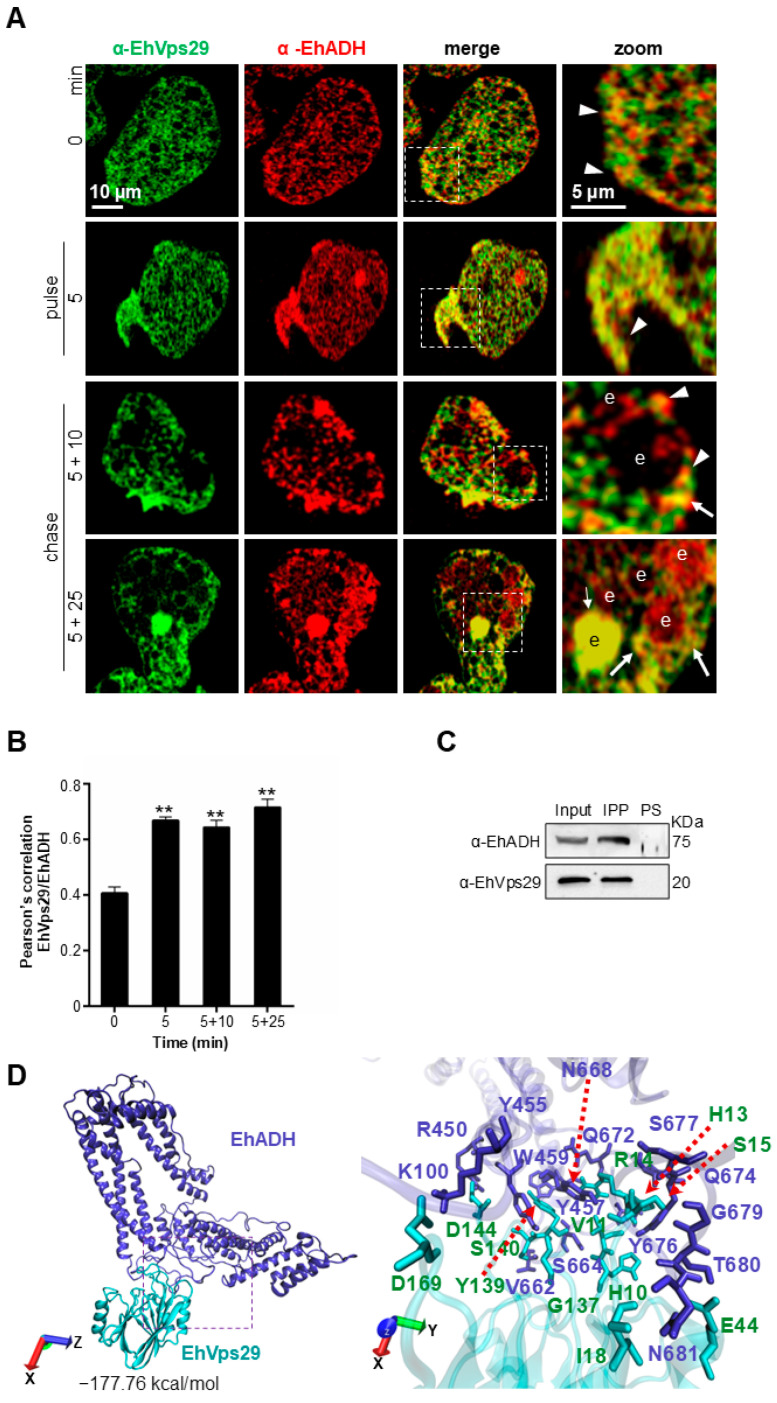
**Localization and interaction of EhVps29 and EhADH (an ESCRT accessory protein) during phagocytosis.** (**A**) Laser confocal microscopy of trophozoites at steady state, 5 min of pulse and pulse–chase after 5, 10 and 25 min, as described. Zoom images: Marked areas by dotted squares. Arrows: Co-localization areas around the ingested erythrocytes. Arrowheads: Co-localization areas in plasma membrane. e: Erythrocyte. (**B**) Pearson’s correlation between EhVps29 and EhADH fluorescence during phagocytosis. Twenty 0.5 µm z-stacks were considered for quantification. ** *p* < 0.01. (**C**) α-EhVps29 antibodies were fixed to G-agarose beads and interacted with trophozoite total extracts, then the immunoprecipitates were probed with α-EhVps29 and α-EhADH antibodies. PS: Preimmune serum. Numbers at right: Molecular weights. (**D**) Molecular docking of EhVps29 and EhADH proteins, obtained by blind docking assays in the ClusPro server. Right: Zoom of the region marked in the square, containing the contact surface between proteins (for details check Table 2). Binding energy is indicated.

First, we observed EhVps29 in the localizations described above (Figure 3) and EhADH in the plasma membrane and cytosol of trophozoites (Figure 5A). Both proteins co-localized at the plasma membrane and cytosol, suggesting their association. Then, during the pulse (5 min) and 15 min of interaction (5 + 10 min), both proteins were mobilized to the phagocytic cups and co-localized (Figure 5A). At 15 min, EhVps29 and EhADH were also found together around phagosomes (Figure 5A, arrowheads). Finally, at 30 min of interaction (5 min pulse + 25 min chase), the main co-localization areas for EhVps29 and EhADH were around or in phagosomes (Figure 5A, arrows). Immunofluorescence results were consistent with the Pearson’s coefficient, which had a value of 0.4 in steady state, 0.68 at the pulse time, 0.65 at 15 min, and 0.73 at 30 min of phagocytosis (Figure 5B).

These co-localization regions may correspond to microdomains for cargo sorting. In *A. thaliana*, the ALIX protein acts as a retromer recruiter to the plasma membrane [31]. This could also be taking place in the amoebic model, leading to the formation of recycling and degradation domains in the phagosomal membranes.

Then, we performed co-immunoprecipitation experiments using the α-EhVps29 antibody coupled to G-agarose beads and interacted with amoebic total extracts. The immunoblots revealed 75-kDa and 20-kDa bands, corresponding to EhADH and EhVps29, respectively (Figure 5C). As our group reported the interaction of EhVps35 and EhADH in pulse and chase experiments (2 and 30 min of interaction) [30], these results suggest that the association between EhVps29 and EhADH could remain and increase during phagocytosis or that this is a dynamic process.

We identified the putative interaction sites between EhVps29 and EhADH by docking analyses, using the EhVps29 crystal and the EhADH 3D structure [43] in blind docking conditions. The global free binding energy for the best pose of EhVps29-EhADH was 177.76 kcal/mol (Figure 5D). The binding region was formed by three salt bridges and 19 hydrogen bonds (Table 2). The interacting residues in EhADH were K100, R450, Y455, Y457, W459, V662, S664, N668, Q672, Q674, Y676, S677, T680, N681, R450, and R663, while the interacting residues in EhVps29 were D169, D144, G137, S140, S15, Y139, H10, H13, R14, V11, S15, E44, I18, and D62 (Figure 5D).

EhADH is an adhesin that, along with the EhCP112 cysteine protease, forms the EhCPADH complex, contributing to the virulence during *E. histolytica* infection and invasion [70,71]. Thus, we do not discard the possibility that EhCP112 could be interacting with EhVps29 or being transported by the retromer. In fact, we have demonstrated that EhADH is recycled by EhVps35 [30].

Altogether, our results revealed the interaction between EhVps29 and representative ESCRT proteins. Further experiments are needed to prove if EhVps29 also interacts with other components from the ESCRT-I and ESCRT-III complexes and to determine whether the interactions are direct or mediated by other proteins.

### 3.6. The Ehvps29 Knockdown Affects EhVps26

The interaction between Vps29 and Vps35 proteins has been associated with CSC stabilization [22,72,73,74]. In *D. melanogaster*, Vps29 is crucial for CSC stability, location, and detachment from the endosomal membrane [22]. In HeLa cells, Vps35 and Vps26 proteins have a loss of 75% and 90% in expression when the *Vps29* gene is knocked out [25].

**Figure 6:**
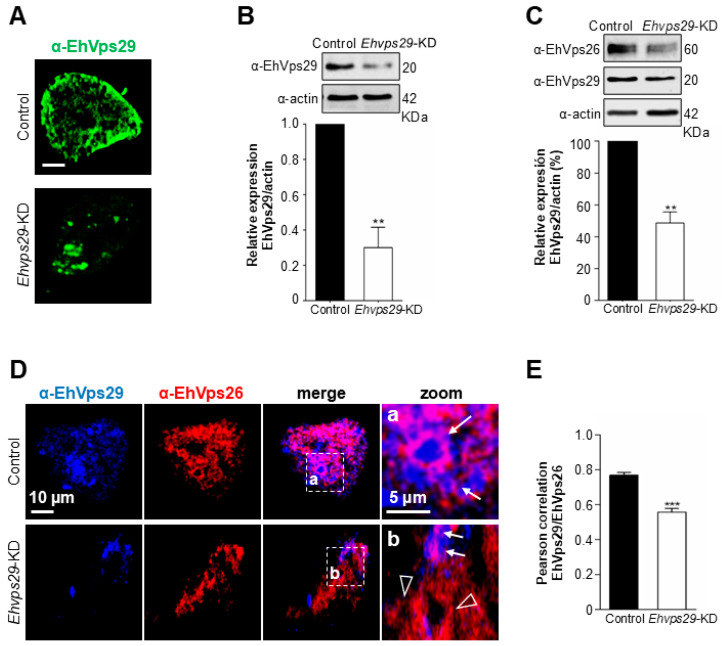
**Knockdown of the Ehvps29 gene in trophozoites and effects in CSC expression.** (**A**) The *Ehvps29* gene was knocked down using dsRNAs. Then, knocked-down cells were observed through confocal microscopy using the α-EhVps29- and FITC-coupled secondary antibodies. (**B**) Total extracts of wild-type (control) and knocked-down trophozoites (*Ehvps29*-KD) were processed for SDS-PAGE and Western blot assays. Representative results from three independent experiments. ** *p* < 0.01. (**C**) Detection of EhVps26 and EhVps29 by Western blot. Trophozoite total extracts of control and *Ehvps29*-KD cells were probed with α-EhVps29 and α-EhVps26 antibodies. The results are representative of three independent experiments. ** *p* < 0.01. (**D**) Detection of EhVps29 (blue) and EhVps26 (red) by immunofluorescence in control and knockdown trophozoites at steady state. Zoom areas: Marked squares (**a**,**b**). Arrows: Vesicles marked by both fluorochromes. Arrowheads: Vesicles marked only by EhVps26. (**E**) Pearson’s correlation of EhVps29 and EhVps26 signals. The plot considered twenty 0.5 µm z-stacks. *** *p* < 0.001.

Here, we investigated how the *Ehvps29* knockdown affects the EhVps26 expression and localization. We knocked down the *Ehvps29* gene by using dsRNAs methods [40]. The *Ehvps29* knocked-down trophozoites (*Ehvps29*-KD population) expressed 70% less EhVps29 protein than control cells, as seen by confocal microscopy and Western blot (Figure 6A,B).

Western blot assays using the α-EhVps26 antibodies showed a 51% decrease in the EhVps26 protein in *Ehvps29*-KD trophozoites with respect to the control (Figure 6C).

Confocal microscopy using the α-EhVps26 and α-EhVps29 antibodies exhibited EhVps26 and EhVps29 co-localizing throughout the cytosol and cytoplasmic vesicle-like structures in wild-type trophozoites (Figure 6D, arrows). In *Ehvps29*-KD cells, both proteins were present in lower levels and co-localized in cumuli (Figure 6D, arrows), although vesicle-like structures only displayed EhVps26 (Figure 6D, arrowheads). The Pearson’s correlation was 0.78 for control and 0.55 for *Ehvps29*-KD cells, corroborating a significant decrease in EhVps26 and EhVps29 co-localization (Figure 6E).

Our results suggest that *Ehvps29* knockdown reduces EhVps26 levels, probably affecting the retromer conformation due to an inadequate stoichiometry.

### 3.7. Ehvps29-KD Trophozoites Display EhVps26 and EhVps29 Mislocalization in Golgi-like Structures

**Figure 7:**
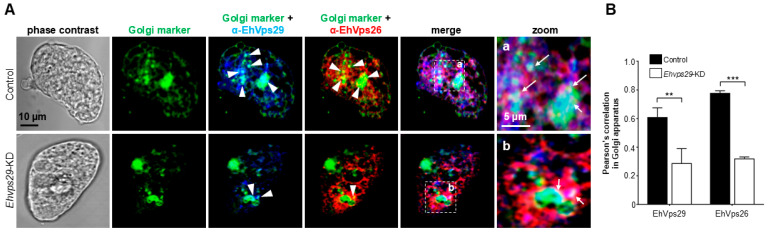
**Localization of EhVps29 and EhVps26 in Golgi-like structures in trophozoites.** (**A**) Confocal microscopy of control and knocked-down cells incubated with NBD C6-ceramide (green), α-EhVps29 (blue) and α-EhVps26 (red) antibodies. Zoom images: Marked squares in (**a**,**b**). Arrows: Co-localization areas of EhVps29 and EhVps26 proteins in the Golgi-like structure. (**B**) Pearson’s correlation between the Golgi marker and EhVps29 or EhVps26. Plot is representative of twenty 0.5 µm z-stack images from control and knockdown cells. ** *p* < 0.01, *** *p* < 0.001.

Previous reports have described the variation in the Golgi apparatus pattern upon knockdown of the Vps35 retromer component [75]. Other groups have found a fragmented organelle and a decrease in CI-M6PR and Wls retromer cargo localization in the Golgi, demonstrating altered retrograde transport [75,76]. In this work, to analyze the retromer localization in the Golgi apparatus by confocal microscopy, we used the NBD-C6 ceramide probe and α-EhVps29 and α-EhVps26 antibodies. We observed the Golgi-like structures (green) in the cytosol of control and *Ehvps29*-KD trophozoites (Figure 7A). In *Ehvps29*-KD trophozoites, the co-localization areas decreased for the Golgi (green) and EhVps29 (blue) (Figure 7A, arrowheads). An akin effect was found in the co-localization of EhVps26 (red) and Golgi (Figure 7A, arrowheads). We also detected fewer co-localization areas for EhVps26, EhVps29, and the Golgi compared to control trophozoites (Figure 7A, arrows). In *Ehvps29*-KD trophozoites, the Pearson’s correlation revealed a 54% and 59% decrease in EhVps29 and EhVps26 co-localization with the Golgi (Figure 7B).

Moreover, since the *Ehvps29* knockdown altered the location of the retromer subunits in the Golgi, one would expect the location of cargo proteins also to be affected. The possible changes in the location of *E. histolytica* retromer cargoes in the absence of retromer components should be investigated in future recycling assays.

### 3.8. Ehvps29 Knockdown Affects Trophozoite Adhesion, Phagocytosis, and Cytopathic Effect

Earlier works have reported the involvement of the retromer in some pathological processes; e.g., in human papillomavirus infection, the presence of SNX27 and the CSC is important for viral DNA transport into the TGN, allowing virus replication [77,78]. In *T. gondii*, the Vps35 protein is crucial for maintaining parasite morphology and virulence factor localization [79]. In addition, the RidL protein from the *Legionella pneumophila* bacterium hijacks Vps29 to allow its intracellular growth [80].

**Figure 8:**
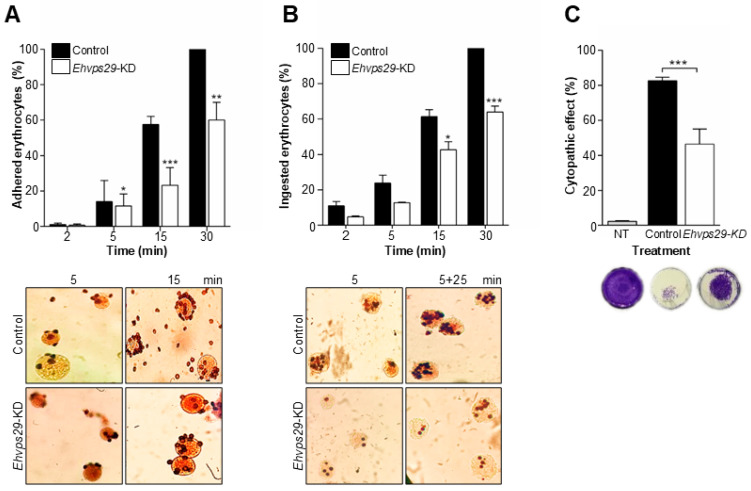
**Effect of *Ehvps29* knockdown in adhesion, phagocytosis and cytopathic damage.** Control and *Ehvps29*-KD trophozoites were incubated at 4 °C (for adhesion) or 37 °C (for phagocytosis) with RBCs for different times, according to the methodology described. Rates of adhesion (**A**) and phagocytosis (**B**). Plots are representative of three independent experiments by duplicated time samples. Images at the bottom are representative of each condition. * *p* < 0.05, ** *p* < 0.01, *** *p* < 0.001. (**C**) Control and knockdown trophozoites were incubated with MDCK cell monolayers for 40 min and monolayer destruction was measured by methylene blue assays as described in Materials and Methods. The plot is representative of two independent experiments considering quadruplicated samples by treatment. *** *p* < 0.01. Images on the right are representative wells. NT: Cells incubated with TYI medium (no trophozoites added).

Here, by using *Ehvps29*-KD trophozoites, we performed adhesion, erythrophagocytosis, and cytopathic effect assays to evaluate the EhVps29 effects on virulence. In adhesion assays, no significant changes were found at 2 min of RBC interaction with *Ehvps29*-KD trophozoites. In contrast, at 5 and 15 min of interaction, these cells adhered 22% and 60% less to RBCs than the control ones, respectively (Figure 8A). At 30 min of interaction, there was a 40% decrease in adhesion rate in *Ehvps29*-KD compared to control trophozoites (Figure 8A).

We also evaluated the rates of phagocytosis. The results showed no significant differences between *Ehvps29*-KD and control trophozoites at 2 and 5 min of phagocytosis (Figure 8B). However, at 15 and 30 min, *Ehvps29*-KD cells ingested 31% and 37% fewer RBCs than the control trophozoites, respectively (Figure 8B). These results suggest the participation of EhVps29 in adhesion and phagocytosis, although we do not know the reason why adhesion is more affected than phagocytosis.

Next, we evaluated the damage caused by both amoebae populations on MDCK monolayers for 40 min. Monolayers incubated with TYI medium were used as a negative control. We found 44% less damage caused by *Ehvps29*-KD trophozoites on MDCK monolayers compared to the control (Figure 8C). It is known that cytopathic damage involves transport and secretion of several virulence factors, mainly cysteine proteases [27,28]; thus, these molecules could also be directly or indirectly affected by the *Ehvps29* knockdown.

In addition, adhesion, phagocytosis, and cytopathic damage involve well-described molecules such as EhADH, EhCP112, and the EhCPADH complex [70,71]. EhADH is a retromer recycling target [30]; hence, we cannot rule out that EhCP112 could also be transported by the retromer. In conclusion, the EhVps29 deficiency may lead to the misregulation of the retromer cargo proteins, including virulence factors.

### 3.9. EhVps29 Interacts with EhCP112 Virulence Factor

The retromer has been involved in the transport and expression of virulence factors. This is the case of the Vps5 protein (SNX dimer), whose knockdown causes a decrease in the expression level of ISG65 and ISG75 transmembrane proteins in *T. brucei* [10]. Furthermore, the knockout of *Vps35* in *T. gondii* leads to the mislocalization of GRA1, ROP2-3, and MIC5 virulence proteins [79]. In addition, EhADH and EhGal-Gal/Nac lectin are cargoes of the *E. histolytica* retromer [30]. These molecules have been widely involved in pathogenicity [70,71,81]. Here, we analyzed the expression and location of EhADH and EhCP112 and their interaction with EhVps29 in *Ehvps29*-KD trophozoites.

**Figure 9:**
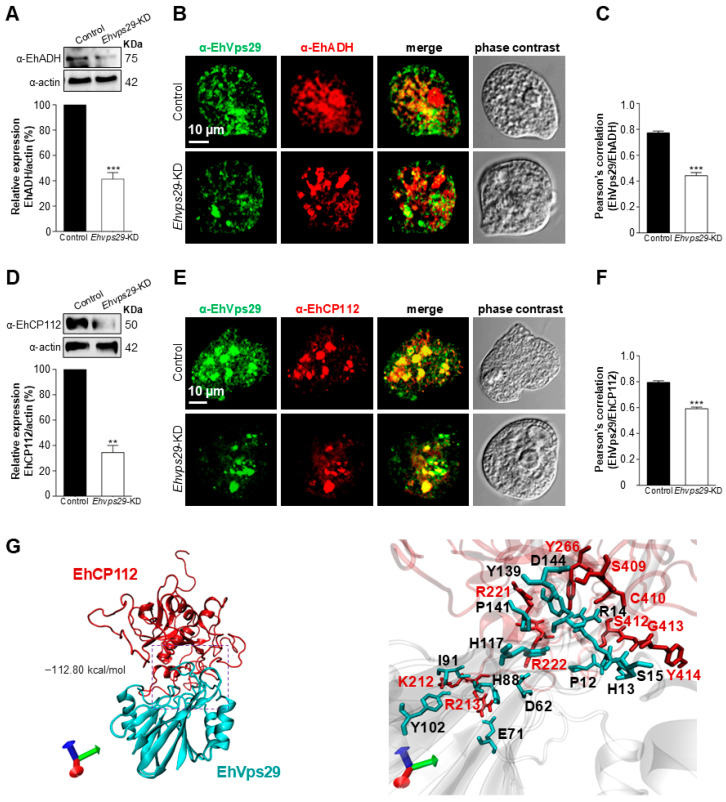
**Detection of EhADH and EhCP112 in control and *Ehvps29*-KD trophozoites.** (**A**) Detection of EhADH in control and knockdown trophozoites by Western blot. Amoebic total extracts were probed with α-EhADH. Actin was used as loading control. Results are representative from three independent experiments. Numbers on the right: Molecular weights. (**B**) Confocal microscopy images of EhVps29 (green) and EhADH (red). Images are representative of three independent experiments. (**C**) Pearson’s correlation of EhVps29 and EhADH. The plots are representative of twenty 0.5 µm z-stacks. ** *p* < 0.01; *** *p* < 0.001. (**D**–**F**) same as (**A**–**C**) but detecting EhCP112. (**G**) Molecular docking of EhVps29 and EhCP112 (for further details see Table 3). The image on the right is a zoom from the marked area in a square. The images were obtained by VMD software. Binding energy is indicated.

First, we performed Western blot assays using amoebic total extracts probed with rat α-EhVps29, rabbit α-EhADH, or rabbit α-CP112 antibodies [70,71]. We found a decrease in EhADH and EhCP112 protein levels in 59% and 65%, respectively, in the *Ehvps29*-KD trophozoites compared to the control (Figure 9A,D). Confocal microscopy images exhibited EhADH and EhVps29 proteins in the plasma membrane and cytoplasmic vacuoles, as described before [70] and above. Both proteins were also observed in cumuli (Figure 9B). EhCP112 was found mainly in cumuli control and *Ehvps29*-KD cells (Figure 9E). Pearson’s correlation scores indicated a 43% loss of co-localization between EhVps29 and EhADH and 25% less co-localization of EhVps29 and EhCP112 in *Ehvps29*-KD versus control trophozoites (Figure 9C,F). These results indicate that EhADH and EhCP112 localization is affected by the *Ehvps29* gene knockdown.

In concordance with the EhVps29 and EhADH interaction, the blind molecular docking analyses showed that EhCP112 also interacts with EhVps29, displaying a binding energy of −112.80 kcal/mol. The interaction site was formed by two salt bridges and 14 hydrogen bonds. The binding residues of EhCP112 were K212, R213, R221, Y266, S409, C410, S412, G413, Y414, R213, and R222, while for EhVps29 they were I91, Y102, E71, H88, Y139, P141, H117, R14, D144, P12, H13, S15, E71, and D62 (Figure 9G, Table 3).

This interaction supports the hypothesis that EhCP112 could be a retromer cargo, as described for other cysteine proteases.

### 3.10. Ehvps29 Overexpression Does Not Alter Rates of Adhesion, Improves Phagocytosis, and Decreases the Cytopathic Effect

To evaluate the EhVps29 protein function, we overexpressed the *Ehvps29* gene. HM1:IMSS trophozoites were transfected with the pNeo-*Ehvps29* or empty pNeo plasmids. Overexpression was induced by the addition of neomycin to trophozoite cultures.

**Figure 10:**
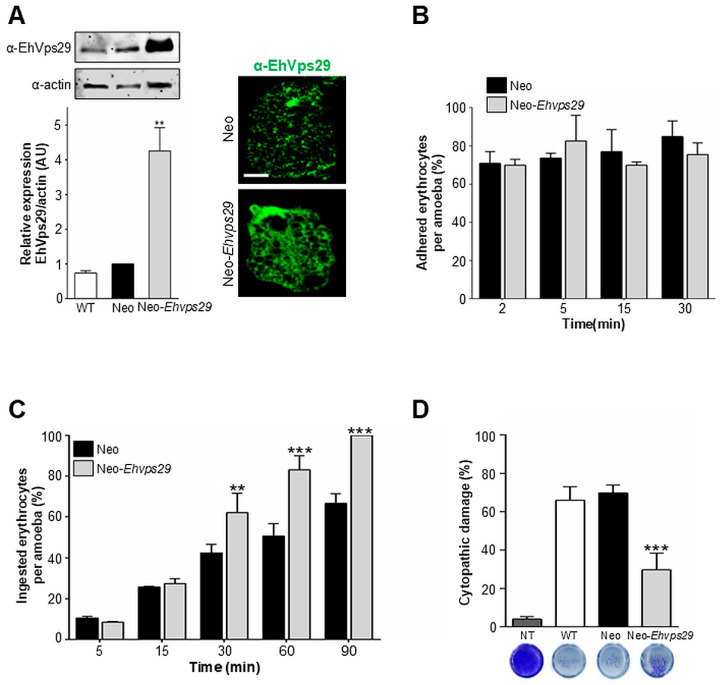
**EhVps29 overexpression in trophozoites and its effects on virulence.** (**A**) Western blot and immunofluorescence assays using α-EhVps29 antibodies. Actin was used as loading control. The results are representative from three independent experiments. WT: Wild-type trophozoites, Neo: Trophozoites transfected with the empty pNeo vector, and Neo-*Ehvps29*: trophozoites transfected with pNeo-*Ehvps29* construct. ** *p* < 0.01. (**B**) Rates of adhesion at 2, 5, 15 and 30 min of trophozoites interaction with RBCs. The plot is representative of three independent experiments by duplicate. (**C**) Rates of phagocytosis in Neo and Neo-*Ehvps29* trophozoites at 5, 15, 30, 60 and 90 min. The plot is representative of three independent experiments by duplicate. ** *p* < 0.01, *** *p* < 0.001. (**D**) Cytopathic effect caused on MDCK cell monolayers. Images at the bottom are representative wells of each condition. NT: Cells incubated with TYI medium (no trophozoites). *** *p* < 0.01. The plot is representative of two independent experiments in quadruplicate.

Western blot and immunofluorescence experiments, using the α-EhVps29 antibody, revealed the overexpression of EhVps29 at least 4-fold in the Neo-*Ehvps29* population compared to Neo trophozoites (Figure 10A). A consistent result was observed by confocal microscopy (Figure 10A).

Then, we analyzed the rates of RBC adhesion of Neo-*Ehvps29* and Neo trophozoites. The rates of adhesion were similar in both populations, as the percentage of adhered RBCs fluctuated between 70 and 80% during all considered interaction times (Figure 10B).

In phagocytosis assays, no significant changes in ingested erythrocytes were observed at 5 and 15 min between Neo-*Ehvps29* and Neo populations (Figure 10C). Nevertheless, at 30, 60, and 90 min, respectively, Neo-*Ehvps29* trophozoites ingested 32%, 40%, and 33% more RBCs than Neo cells (Figure 10C). It is possible that protein overexpression causes the overloading of a cellular transport pathway, impeding proteins from being carried to their fate, as reviewed in [82,83].

Cytopathic effect assays using Neo-*Ehvps29* showed a 58% damage decrease on MDCK cells regarding Neo parasites (Figure 10D). Here, it is possible that the system reaches a saturation plateau, causing the impairment of the whole CSC, then altering cargo virulence factors’ location and function. We hypothesize that, as seen by others [27,28], the cysteine protease transport could be affected by the *Ehvps29* gene overexpression.

In this regard, phagocytosis is a process triggered by the binding of a particle to a cell surface molecule, which leads to cytoskeletal rearrangement that ultimately allows phagosome formation [84]. In this work, we suggest that distinct amoebic molecules may be differentially activated in response to variant host cell stimuli, such as RBCs and MDCK cells. This dissimilar activation could account for the observed variations in adhesion, phagocytosis, and cytopathic effect, even when the same Neo and Neo-*Ehvps29* amoebae are used.

Alterations in the expression and function of proteins involved in actin-cytoskeleton arrangement could further explain differences in pathogenic events found here. In this case, we do not exclude the possibility that the EhCP112 protease, and even other proteases, could be affected by the *Ehvps29* overexpression due to defects in intracellular trafficking. Similarly, other CSC cargo, such as actin-binding proteins (ABPs) or calcium-binding proteins (e.g., EhCaBP6 [57]), may exhibit altered transport, impairing actin polymerization and, consequently, phagosome maturation.

Moreover, Rab GTPases regulate phagosome maturation, and the *E. histolytica* retromer has been identified as a Rab7A effector [27]. This relationship suggests that overexpression of retromer components or Rab7A could disrupt Rab7A–retromer-mediated processes, thereby affecting the overall transport and functionality of the phagocytic machinery. A previous work reported cytotoxic effect impairment under EhRab7A overexpression [27].

As previously reviewed [84], the identification of key cell signaling effectors that regulate phagocytosis points out the complexity of the ingestion mechanisms employed by this parasite, which suggests that upon *Ehvps29* overexpression, only some phagocytosis- and cytopathic effect-related proteins are affected. It is necessary to further investigate the effect of *Ehvps29* overexpression on virulence-related molecules, and to clarify the mechanisms underlying the differential effects observed in phagocytosis and cytopathic activity.

## 4. Discussion

The protozoan retromer and its interactions with other molecular complexes remain poorly characterized. This work aimed to elucidate the role of EhVps29 in *E. histolytica*, focusing on its roles in virulence and vesicular trafficking.

The presence of EhVps29 in the plasma membrane, Golgi, cytosol, and intracellular and extracellular vesicles matches with findings in other systems [27,29,30,56,60], and its location in tubular structures resembles those described in *Chlamydomonas reinhardtii* and *Chaetomium termophilum* [54]. In addition, we found EhVps29 in the nucleus of trophozoites, although this protein does not contain an NLS and belongs to the metallophosphatase protein family [28]. In this regard, metallophosphatases such as PPM1D (wild-type p53-induced protein phosphatase 1, or Wip1) regulate DNA damage response and nuclear transcription [85]. In addition, it has been described that the Vps26, Vps35, and SNX3 retromeric proteins are temporarily translocated to the nucleus to allow the STAT3 transcription factor function [86]. It is possible that EhVps29 targets the nucleus and accomplishes some functions yet unknown.

EhVps29 was also located in MVBs (Figure 1C, panel e; Figure 3B, panel b3). This may reflect retromer-mediated cargo transport or EhVps29 degradation in these organelles. Vps26, Vps35, Vps29, and SNX1 have been found in MVBs in other systems [87,88], although their precise roles remain unknown. The EhVps26 and EhVps29 co-localization in the plasma membrane, cytosol, vesicles, and Golgi (Figure 2B) strongly suggests that the CSC could be assembled through the endocytic pathway.

During phagocytosis, EhVps29 was mobilized to the phagocytic cups and phagosomes (Figure 3, Figure 4A, and Figure 5A), suggesting that this protein has a role in target cell engulfment. EhVps26, EhVps35, and EhSNX3-like proteins have also been found in phagocytic cups and phagocytosis-related vacuoles [27,28,29,30,53], reinforcing that CSC is in the plasma membrane through endosome maturation.

The role of EhVps29 in phagocytosis is strengthened by its interaction with the ESCRT machinery, which is highly involved in this event [36,38,41]. In this paper, we described that EhVps29 interacts with EhVp36 (ESCRT-II) and EhADH (ESCRT-accessory protein) as phagocytosis progresses, showing that the retromer associates with the early and late ESCRT components. In both cases, the proteins were relocated towards RBCs’ contact points. The interaction between Vps27 (ESCRT-0) and Vps26, Vps17, SNX3, and the iron transporter Fet3-Ftr1 in *S. cerevisiae* endosomes was described before [33], strengthening a close relationship between ESCRT and retromer complexes.

EhVps26 levels and its localization in the Golgi were affected when the EhVps29 protein was knocked down (Figure 6C and Figure 7), in agreement with other reports where CSC members’ expression is altered after knocking out *Vps26*, *Vps35,* or *Vps29* genes in human cells and *A. thaliana* [26,76,89]. In our model, EhVps26 and EhVps29 mislocalization in the Golgi could have been caused by knocking down *Ehvps29*, which probably led to a CSC permanent attachment to endosomes, disturbing the CSC location and cargo destination, as seen by others [22].

Phagocytosis and cytopathic effect were distressed after the *Ehvps29* knockdown or overexpression (Figure 8 and Figure 10), suggesting that certain molecules participating in these processes were affected. We hypothesized that altering EhVps29 expression caused CSC disturbance, involving a dysfunctional sorting and location of molecules that participate in adhesion, phagocytosis, and cytopathic damage, such as the EhADH adhesin and the EhCP112 cysteine protease [37,69,70,81,90]. *Ehvps29* overexpression possibly produced the overloading of a cellular transport pathway, impeding proteins from being carried to their fate, as reported [82,83]. Hence, in knocked-down *E. histolytica* trophozoites, EhADH, EhCP112, and other molecules could be trapped in endosomes, vesicles, or lysosomes, preventing their proper sorting and secretion to damage the host cells.

**Figure 11:**
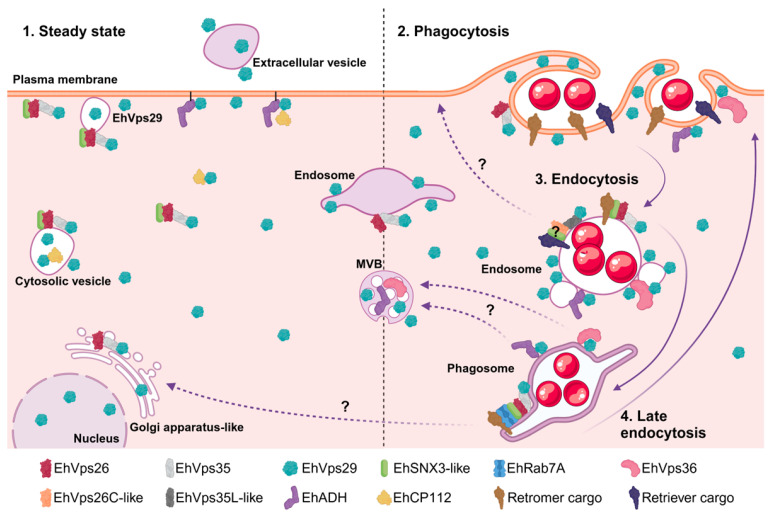
**EhVps29 working model.** Localization of EhVps29 and the retromer in trophozoites at steady state and phagocytosis. 1. EhVps29 and retromer cellular localization in the plasma membrane, cytosol, vesicles, Golgi, MBVs, and nucleus in steady state conditions. 2. EhVps29 and the retromer are recruited to the phagocytic cups, along with EhVps36 and EhADH. 3. Recruitment of EhVps29 and the retromer to endosomes, phagosomes and adjacent vesicles. This could be the stage where the retriever complex (if expressed in amoeba) sorts cargoes for recycling to the plasma membrane. 4. On late endosomes, the retromer cargoes are sorted for transport to the plasma membrane or to the Golgi network. This step could also imply the transport of EhVps29 towards the MVBs. Question marks are added to indicate putative transport pathways not yet described in *E. histolytica*. Created in BioRender. Bañuelos, C. (2025) https://BioRender.com/gd9f8nl (accessed on 21 February 2025).

As a working model, we propose that the presence of EhVps29 in *E. histolytica* trophozoites (Figure 11) increases after RBC stimulation, evidencing a dynamic process of the retromer. It is possible that the full CSC could be assembled in the Golgi and plasma membrane since basal conditions (Figure 2B and Figure 7A). EhVps36 and EhADH movement towards the phagocytic cups suggests that cargo sorting starts from the early stages of phagocytosis. Once phagosomes are formed, cargo-enriched domains are constituted, preparing proteins for sorting and transport (Figure 11). In these organelles, a putative retriever complex could act to sort cargoes as well, transporting them to the plasma membrane.

As a summary, the evidence shown in this work demonstrated that EhVps29 is involved in adhesion, phagocytosis, and cytopathic effect, probably through the transport mediated by the retromer and/or the retriever complexes. In addition, the EhVps29 is important for the CSC formation and localization in the Golgi-like organelle. In fact, EhVps29 influences EhVps26 expression and localization in the Golgi. Additional evidence showed an interaction between EhVps29 and the ESCRT members (EhVps36 and EhADH).

Although the retriever complex has not been experimentally characterized, preliminary in silico analyses predicted single-copy genes encoding EhVps26C-like (EHI_010730) and EhVps35L-like (EHI_000470) proteins in amoebae, suggesting the presence of a retriever-like complex in this parasite. Therefore, EhVps29 may be a component of either the retromer or a retriever-like complex. The detection of EhVps29 in MVBs 30 min after phagocytosis suggests that the cargo-selective complex (CSC) may participate in cargo trafficking toward degradation, or that CSC components themselves are targeted for degradation via the MVB pathway. Further experimental studies are required to elucidate the functional interplay between retromer and ESCRT complexes.

## Figures and Tables

**Table 1:** Interactions between EhVps29 and EhVps36.

	EhVps29	EhVps36	Distance (Å)		EhVps29	EhVps36	Distance (Å)
**1**	H13	R160	2.70	**8**	T116	Y233	2.84
**2**	H13	D161	2.85	**9**	T116	Q229	2.76
**3**	R14	E165	2.96	**10**	T116	Q229	2.93
**4**	H88	E208	2.93	**11**	K118	Y233	2.66
**5**	W93	T228	2.79	**12**	L119	Y235	2.91
**6**	H115	L231	2.72	**13**	Y139	N169	2.96
**7**	T116	Q232	2.93	**14**	R14	E165	2.71 *

* Salt bridges. Distances are shown in Å.

**Table 2:** Detailed interactions between EhVps29 and EhADH.

	EhVps29	EhADH	Distance (Å)		EhVps29	EhADH	Distance (Å)
**1**	D169	K100	2.58	**12**	V11	Y676	2.73
**2**	D144	R450	2.99	**13**	H13	Y676	2.90
**3**	G137	Y455	2.85	**14**	S15	S677	2.89
**4**	S140	Y455	2.93	**15**	S15	G679	2.81
**5**	S15	Y457	2.89	**16**	E44	T680	2.79
**6**	Y139	W459	2.81	**17**	E44	N681	3.04
**7**	S140	V662	2.75	**18**	I18	N681	3.01
**8**	H10	S664	3.08	**19**	E44	N681	2.97
**9**	H13	N668	3.19	**20**	D169	K100	2.58 *
**10**	R14	Q672	2.65	**21**	E44	R450	2.76 *
**11**	H13	Q674	2.75	**22**	D62	R663	2.75 *

* Salt bridges. Distances are shown in Å.

**Table 3:** Detailed interactions between EhVps29 and EhCP112.

	EhVps29	EhCP112	Distance (Å)		EhVps29	EhCP112	Distance (Å)
**1**	I91	K212	2.60	**9**	D144	S409	2.89
**2**	Y102	K212	2.69	**10**	R14	C410	2.71
**3**	E71	R213	2.70	**11**	P12	S412	2.71
**4**	H88	R213	2.90	**12**	H13	S412	2.97
**5**	Y139	R221	2.73	**13**	H13	G413	2.81
**6**	P141	R221	3.09	**14**	S15	Y414	2.73
**7**	H117	R222	2.90	**15**	E71	R213	2.70 *
**8**	R14	Y266	2.68	**16**	D62	R222	2.65 *

* Salt bridges. Distances are shown in Å.

## Data Availability

The original contributions published in this study are included in the article. Further inquiries can be directed at the corresponding authors.
